# Label‐Free Metal‐Oxide Transistor Biosensors for Metabolite Detection in Human Saliva

**DOI:** 10.1002/advs.202306038

**Published:** 2024-02-21

**Authors:** Abhinav Sharma, Hendrik Faber, Wejdan S. AlGhamdi, Dipti Naphade, Yen‐Hung Lin, Martin Heeney, Thomas D. Anthopoulos

**Affiliations:** ^1^ King Abdullah University of Science and Technology (KAUST) KAUST Solar Center (KSC) Thuwal 23955–6900 Saudi Arabia; ^2^ Department of Electronic and Computer Engineering The Hong Kong University of Science and Technology Clear Water Bay Kowloon Hong Kong; ^3^ Photon Science Institute, Henry Royce Institute Department of Electrical and Electronic Engineering, The University of Manchester Manchester M13 9PL United Kingdom

**Keywords:** human saliva, metabolite sensing, metal oxide transistor, non‐invasive sensor, transistor biosensor

## Abstract

Metabolites are essential molecules involved in various metabolic processes, and their deficiencies and excessive concentrations can trigger significant physiological consequences. The detection of multiple metabolites within a non‐invasively collected biofluid could facilitate early prognosis and diagnosis of severe diseases. Here, a metal oxide heterojunction transistor (HJ‐TFT) sensor is developed for the label‐free, rapid detection of uric acid (UA) and 25(OH)Vitamin‐D3 (Vit‐D3) in human saliva. The HJ‐TFTs utilize a solution‐processed In_2_O_3_/ZnO channel functionalized with uricase enzyme and Vit‐D3 antibody for the selective detection of UA and Vit‐D3, respectively. The ultra‐thin tri‐channel architecture facilitates strong coupling between the electrons transported along the buried In_2_O_3_/ZnO heterointerface and the electrostatic perturbations caused by the interactions between the surface‐immobilized bioreceptors and target analytes. The biosensors can detect a wide range of concentrations of UA (from 500 nm to 1000 µM) and Vit‐D3 (from 100 pM to 120 nm) in human saliva within 60 s. Moreover, the biosensors exhibit good linearity with the physiological concentration of metabolites and limit of detections of ≈152 nm for UA and ≈7 pM for Vit‐D3 in real saliva. The specificity is demonstrated against various interfering species, including other metabolites and proteins found in saliva, further showcasing its capabilities.

## Graphical abstract

All solid‐state transistor biosensors based on metal oxide heterojunction channels that are able to detect uric acid and 25(OH)Vitamin‐D3 (Vit‐D3) in human saliva are demonstrated. The sensors are simple to manufacture and combine rapid response with selectivity toward the specific metabolites and good operational stability.

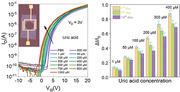

## 1 Introduction

Metabolites play a major role in the human body by participating in various metabolic pathways that contribute to several biological and physiological processes.^[^ ^1^, ^2^ ^]^ The monitoring of metabolites for the early diagnosis of anomalous health conditions has the potential to enable the implementation of early intervention treatments and adjustment of precision nutrition to counteract or prevent serious conditions. Uric acid (UA) plays an important role in various biological functions in humans and is the main end product of purine metabolism in the human body.^[^ ^3^, ^4^ ^]^ The normal UA concentration in blood for healthy individuals varies between 100 and 400 µM.^[^ ^5^ ^]^ However, studies found the UA concentration in saliva of healthy individuals is lower than in their blood and it typically ranges from 30 to 180 µM,^[^ ^6^, ^7^ ^]^ while patients with hyperuricemia observed exceed UA concentrations up to 1 mM in saliva.^[^ ^8^ ^]^ According to several studies, there is a significant linear correlation between the level of UA in saliva and blood serum/plasma.^[^ ^6^, ^9^, ^10^, ^11^, ^12^, ^13^ ^]^ Abnormal UA levels in saliva are indicators of many clinical disorders, such as gout, hyperuricemia, hypertension, cancer, type 2 diabetes, kidney dysfunction, and metabolic disorders etc.^[^ ^13^, ^14^, ^15^, ^16^, ^17^, ^18^, ^19^, ^20^ ^]^ Thus, the determination of UA in saliva is crucial for the diagnosis and treatment of diseases associated with abnormal UA levels and has the potential to replace blood and urine tests due to its non‐invasive sample collection.^[^ ^21^, ^22^ ^]^ While commercially available UA meters offer portability, rapid results, and quantitative measurements with good accuracy via enzymatic‐electrochemical detection, their reliance on invasive finger prick blood collection poses a significant barrier to patient comfort and compliance, particularly for frequent monitoring. In contrast, colorimetric test strips offer a simpler and cost‐effective approach for UA detection in urine samples. However, their significantly lower sensitivity hinders their ability to reliably detect and quantify low UA concentrations, especially in the early stages of gout. Owing to these shortcomings, electrochemical sensors have been mainly developed for the detection of UA levels from non‐invasive fluids (i.e., sweat, saliva, urine). Kim et al. reported an integrated wireless mouthguard biosensor for real‐time monitoring of UA levels in saliva.^[^ ^23^ ^]^ Yang et al. 2020, proposed a CO_2_ laser‐engraved graphene biosensor for ion‐selective electrochemical detection of UA in sweat^[^ ^24^ ^]^ and Kulyk et al. reported a CO_2_ laser‐induced graphene for non‐enzymatic detection of UA in urine.^[^ ^25^ ^]^ Paper‐based microfluidic analytical devices (µPADs) were used for electrochemical and colorimetric detection of UA in urine.^[^ ^26^, ^27^ ^]^ There is an urgent need for portable POC technologies that are sensitive enough and capable of quantifying low concentrations of metabolites.

Vitamin D3 (also known as “calciferol”) is an essential micronutrient and metabolite that plays a vital role in cell and growth development of skeletal and non‐skeletal tissues.^[^ ^28^, ^29^ ^]^ It promotes bone metabolism, homeostasis of hormones and multiple ion (calcium, magnesium, and phosphate) absorption in the intestine, and kidneys.^[^ ^30^, ^31^ ^]^ Vitamin D deficiency plays a significant role in the occurrence of musculoskeletal diseases, obesity, type 2 diabetes and metabolic disorders.^[^ ^32^, ^33^, ^34^ ^]^ Recent studies suggest that the intake of vitamin D is crucial for the prevention and treatment of COVID‐19.^[^ ^35^, ^36^, ^37^ ^]^ In recent years, Vitamin D deficiency has become a public health problem, with an estimated 1 billion people globally deficient due to infectious illnesses, lack of sunlight, or an unhealthy diet.^[^ ^38^ ^]^ Measuring the level of 25‐hydroxy vitamin D3 (25(OH)Vit‐D3) in serum is the most common way to determine the vitamin D status in the blood stream. A deficiency of 25(OH)Vit‐D3 has been defined as levels of < 50 nm, and the optimum level is between 75 to 250 nm, with levels >250 nm considered to be toxic.^[^ ^39^, ^40^ ^]^ Recent studies have observed a positive linear correlation between saliva and serum levels of Vit‐D3 measured by liquid chromatography combined with mass spectroscopy (LC–MS),^[^ ^39^, ^41^, ^42^ ^]^ indicating that the detection of Vit‐D3 in saliva has excellent potential for non‐invasive detection method for monitoring Vitamin D levels.^[^ ^43^ ^]^ Clarke et al. (2019) reported that a concentration of ∼50 pM of 25(OH)D3 in saliva is likely to correspond to serum levels of 50 nm, which may serve as a cut‐off value for identify vitamin D deficiency.^[^ ^39^ ^]^ However, salivary diagnostics of UA with concentrations (≈30 to 180 µM) and Vit‐D3 (≈50 pM to 20 nm) are still challenging because the levels of these metabolites in saliva are significantly lower compared to blood and thus require sophisticated instruments and trained operators.

Saliva is a vital biofluid that contains numerous clinical biomarkers including metabolites, proteins, bacteria, ions and viruses. It can be collected non‐invasively, therefore making it easily accessible for early diagnosis and prognosis of several diseases through monitoring of specific metabolites.^[^ ^44^, ^45^ ^]^ For example glucose levels in saliva can be monitored for diabetic persons.^[^ ^46^ ^]^ Saliva is readily available in large volumes and can be collected repeatedly over time. A healthy individual can be produced between 500 and 1500 mL day^−1^ at a rate of 0.3 mL min^−1^.^[^ ^47^ ^]^ In comparison, the non‐invasive collection of other biofluids, such as sweat, tears, urine, and interstitial fluids, can be more challenging and cause more discomfort to the patients due to various factors.^[^ ^48^, ^49^ ^]^ As a result, using saliva as a diagnostic fluid is a preferential pathway for monitoring of disease progression and therapeutic decisions. Various standard clinical analytical methods including high‐performance liquid chromatography (HPLC), LC–MS, mass spectrometry, spectrophotometry, immunoassays (chemiluminescense, enzyme‐linked immunosorbent assay, radioimmunoassay, etc.) are employed for determination of UA^[^ ^50^, ^51^, ^52^, ^53^, ^54^ ^]^ and Vit‐D levels.^[^ ^55^, ^56^, ^57^ ^]^ However, complex sample preparation, high cost, slow preparation, and requirements for skilled technicians are the major drawbacks of these analytical methods which render them unsuitable for point‐of‐care testing (POCT). On the other hand, electrochemical, colorimetric, and surface plasmon resonance (SPR) methods have been used to detect uric acid and vitamin D mainly in blood samples. Additionally, to date there are only a few studies that focus on electrochemical detection of UA and Vit‐D in non‐invasive fluids, e.g. in human urine^[^ ^25^, ^27^ ^]^ or sweat.^[^ ^58^, ^59^, ^60^ ^]^ When it comes to the detection in saliva, Shi et al. 2020 reported an electrochemical biosensor for the detection of uric acid using multiwall carbon nanotube (MWCNTs) modified screen‐printed carbon electrodes^[^ ^5^ ^]^ and Park et al. 2022, reported a sandwich‐type electrochemical aptasensor to detect Vit‐D.^[^ ^43^ ^]^ Therefore, the development of simultaneously label‐free rapid quantification and electrical detection for UA and Vit‐D3 in accessible biofluids is still a work‐in‐progress.

Solid‐state thin‐film transistors (TFTs) have been proposed as a highly promising bio‐sensing platform due to their ease of miniaturization, robustness, amenability for scale‐up, intrinsic amplification, and potential for rapid and label‐free detection.^[^ ^61^, ^62^, ^63^, ^64^, ^65^ ^]^ These advantages make TFTs an ideal technology for the development of medical diagnostic devices for point‐of‐care testing when compared to other bio‐sensing platforms. Additionally, solution processing of metal‐oxide materials is a cost‐effective alternative to more sophisticated vacuum‐based deposition techniques such as RF sputtering and pulsed‐laser deposition. Despite its simplicity, solution processing enables uniform thin film deposition with precise control over the chemical composition of the channel layer and allows low‐cost, scalable metal‐oxide TFTs with good charge carrier mobility.^[^ ^66^, ^67^, ^68^, ^69^, ^70^, ^71^, ^72^, ^73^ ^]^ Moreover, the approach enables interface engineering for the formation of well‐defined and ultra‐thin hetero‐interfaces between different oxide layers using suitable precursor chemistry, enabling the development of pseudo‐low‐dimensional label‐free biosensors. Recent years have witnessed a renewed interest in the use of field‐effect transistor (FET) as the active biosensing element for highly sensitive biological detection.^[^ ^62^, ^63^, ^74^, ^75^ ^]^ To this end, metal‐oxide transistors offer flexible surface chemistry for monitoring various biomolecules^[^ ^61^, ^64^ ^]^ and offer an alternative sensing platform for POCT due to their good performance and potentially low cost.^[^ ^76^, ^77^, ^78^, ^79^ ^]^ Despite the recent rapid progress, however, reports on TFT‐based biosensors for the non‐invasive detection of UA and Vit‐D3 metabolites, remain scarce.

Herein, we developed heterojunction metal oxide thin‐film transistor (HJ‐TFT)‐based microarrays for the simultaneous and label‐free detection of UA and Vit‐D3 in human saliva. The discrete HJ‐TFTs feature ultra‐thin, solution‐processed In_2_O_3_/ZnO channels with a tri‐channel geometry. The bilayer channel configuration combines high electron mobility with good on/off current ratios and excellent operational stability. These beneficial characteristics are attributed to the formation of a quasi‐2D electron‐gas‐like system at the vicinity of the oxide heterointerface driven by the conduction band offset between In_2_O_3_ and ZnO sublayers.^[^ ^66^, ^67^, ^68^, ^70^, ^80^ ^]^ The central area of the channel in each HJ‐TFT, termed sensing channel, was functionalized with either uricase enzyme or (25(OH)Vit‐D3) antibody using suitable coupling chemistry, to enable selective detection of UA and Vit‐D3 in PBS and human saliva, respectively. The multiplex microarrays showed high specificity and sensitivity towards the two metabolites with LODs of ≈152 nm and ≈7 pM in real saliva for both UA and Vit‐D3, respectively, and fast response times of <60 s.

## 2 Results and Discussion

### 2.1 Surface Characterization

**Figure 1:**
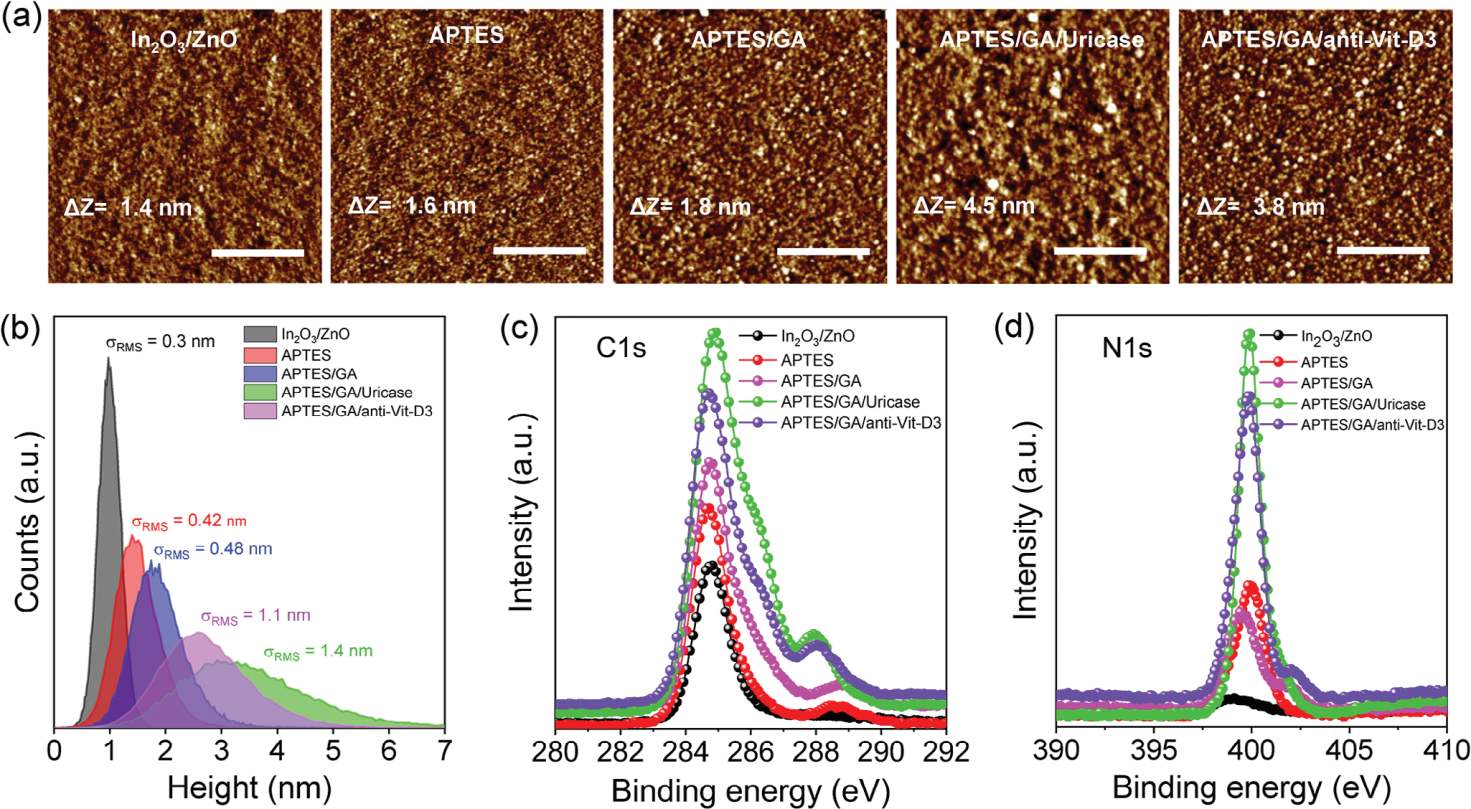
a) AFM topography images of In_2_O_3_/ZnO sensing surfaces before and after each modification scale bar = 500 nm). The corresponding peak‐to‐peak height difference ΔZ) and root mean square surface roughness σ_RMS_) were obtained from AFM image analysis. b) Height histogram extracted from the AFM data for each sequential modification. The C1s XPS spectra c) and N1s peak d) of In_2_O_3_/ZnO before and after each surface modification on a Si/SiO_2_ substrate.

To verify the surface modification of HJ‐TFT microarrays with the different molecules and bioreceptors, several techniques were employed. Static water contact angle (SCA) measurements were used to assess changes in wettability before and after functionalization of APTES/GA layer and bioreceptors. Atomic force microscopy (AFM) was carried out to measure surface topography and roughness, and X‐ray photoelectron spectroscopy (XPS) was used to investigate the surface's chemical composition and bonding states. The SCA measurements after each modification step are summarized in Figure S1 (Supporting Information). The metal‐oxide surface without modification was hydrophilic, with a contact angle of 39° attributed to the presence of ─OH groups. The APTES modified surface showed the highest increase in hydrophobicity, with a contact angle of 87° due to the presence of NH_2_ groups. The contact angle only slightly decreased to 82° after surface modification with GA, and further decreased to 78° and 75° after uricase enzyme and 25(OH)Vit‐D3 antibody, respectively. AFM measurements show the modified sensing surface after each sequential functionalization step (**Figure** **1**a,b). The deposited In_2_O_3_/ZnO exhibits the lowest peak‐to‐peak height (ΔZ) of 1.4 nm with a root‐mean‐square roughness (σ_RMS_) value of 0.3 nm. Moreover, the surface roughness increased after further modification with APTES (ΔZ = 1.6 nm, σ_RMS_ = 0.42 nm) as well as GA (ΔZ = 1.8 nm, σ_RMS_ = 0.48 nm), and reached its value after immobilization of the uricase enzyme (ΔZ = 4.5 nm, σ_RMS_ = 1.4 nm) and 25(OH)Vit‐D3 antibody (ΔZ = 3.8 nm, σ_RMS_ = 1.1 nm) respectively.

Additionally, chemical bonding was confirmed by XPS analysis after each modification step. The XPS data were collected from the surface of a thin film of In_2_O_3_/ZnO. The combined C1s XPS spectra for all steps are shown in Figure 1c, where the incremental increase of the C─C peak intensities at 284.6 eV indicates the presence of additional carbon atoms introduced from each subsequent modification. The individually fitted C1s spectra demonstrate the bonding of functional groups such as C─C (284.6 eV), carbon‐oxygen and nitrogen C─O/C─N (285.8 eV), and additional peaks of C≐O (288.4 eV) and O≐C─O (289.2) that appear after modification with APTES (Figure S2a,b, Supporting Information). The combination of two peaks at C≐O/O≐C─O (at 288.7 and 288.1) appeared after the modification with glutaraldehyde, uricase enzyme and 25(OH)Vit‐D3 antibody (Figure S2c,d,e, Supporting Information). Furthermore, the combination of two peaks at C─O/C─N groups (285.9 eV) indicates the introduction of nitrogen atoms after each immobilization (Figure S2d,e, Supporting Information) (Jung et al. 2013).^[^ ^81^ ^]^ This is also evident in the N1s XPS spectra in Figure 1d, where the intensities of the C─N peak at 399.8 eV increase after the introduction of APTES and further increase after uricase enzyme and 25(OH)Vit‐D3 antibody immobilization. This is attributed to amine groups (─NH_2_) present in the enzyme and antibody structure. The combined results of AFM, SCA, and XPS clearly indicate the successful immobilization of either enzymes or antibodies on the individual In_2_O_3_/ZnO channel surface.

### 2.2 Electrical Characterization of Surface‐Modified Transistors and Microarrays

**Figure 2:**
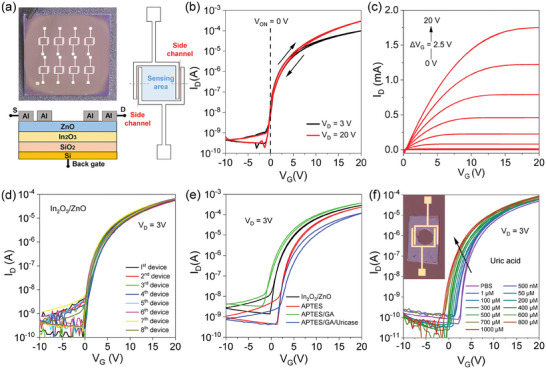
a) A photograph of the tri‐channel HJ‐TFT microarray fabricated on a Si/SiO_2_ substrate and top‐view as well as cross‐sectional schematics. Electrical performance of the bare In_2_O_3_/ZnO) HJ‐TFTs: b) Transfer characteristics under different V_D_ bias, showing a turn‐on voltage close to zero V_ON_ = 0 V). c) Output characteristics of the In_2_O_3_/ZnO HJ‐TFT. d) Multiple transfer characteristics forward and reverse sweeps) of 8 individual HJ‐TFTs. e) Transfer characteristics of the In_2_O_3_/ZnO HJ‐TFT microarray measured before and after each functionalization with APTES, APTES/GA, and APTES/GA/Uricase enzyme. f) Transfer characteristics forward sweeps) for a HJ‐TFT measured at V_D_ = 3 V in the presence of a buffer solution 1 mM PBS) containing uric acid at different concentrations 500 nm to 1000 µM). During the sensing experiment, only the central sensing area was exposed to the analyte. This was guaranteed by attaching a thin layer of PDMS with a round cut‐out acting as a well, as indicated in the inset.

The layout and electrical characteristics of the In_2_O_3_/ZnO HJ‐TFTs microarrays are presented in **Figure** **2**. A photograph of the microarray chip with 8 HJ‐TFTs and schematic diagrams of a single HJ‐TFT (top view and cross‐section) are illustrated in Figure 2a. Each HJ‐TFT consists of two regular side channels and a middle channel, which serves as the sensing area that can be modified with the specific bioreceptors (enzyme or antibody) to detect target metabolites. The transfer and output characteristics of a representative In_2_O_3_/ZnO HJ‐TFT, in the initial state before any surface functionalization, are presented in Figure 2b,c, respectively. The transfer characteristic shows the relationship between the drain current (I_D_) and gate voltage (V_G_), while the output characteristic shows the relationship between I_D_ and drain voltage (V_D_). The In_2_O_3_/ZnO HJ‐TFTs have potential to detect low concentrations of DNA and protein, due to their excellent electron field‐effect mobility (>22 cm^2^ V^−1^ s^−1^) and high on/off ratio (>108) as reported in previous study by Lin et al., (2022).^[^ ^70^ ^]^ In addition, the HJ‐TFTs exhibits negligible device‐to‐device performance variability, which is a crucial aspect for biosensing applications. Figure 2d displays the transfer curves (including forward and backward voltage sweeps) for 8 tri‐channel In_2_O_3_/ZnO HJ‐TFTs in a microarray, demonstrating the highly consistent operating behavior of the individual devices. The high stability of the device performance is indicative of the high quality of the device fabrication process and the robustness of the device design.

Figure S3 (Supporting Information) displays 15 repeated cycles of transfer curves from a single, as‐prepared, HJ‐TFT. The device exhibits consistent electrical characteristics in all measurement cycles without noticeable bias stressing, indicating high operational stability. The good stability is attributed to the unique tri‐channel geometry and the formation of a heterointerface between the In_2_O_3_ and ZnO layers.^[^ ^46^, ^70^ ^]^ The heterointerface facilitates strong coupling between the electrons transported along the buried In_2_O_3_/ZnO heterointerface and the electrostatic perturbations caused by the interactions between bioreceptors and target analytes. The changes in transfer characteristics were measured before (bare surface) and after each successive modification with APTES, GA, and the immobilization of uricase enzyme (Figure 2e), with anti‐25(OH)Vit‐D3 antibody (Figure S4a, Supporting Information) on the In_2_O_3_/ZnO surface. The results show that the channel current (I_D_) decreases for each subsequent modification step, indicating a successful interaction between the functionalized molecules and the oxide surface. The uricase enzyme modified HJ‐TFT exhibit robust operation with stable transfer characteristics even after 120 repeated measurement cycles (Figure S5, Supporting Information).

### 2.3 Sensitivity and Real‐Time Biosensing Measurements

To determine the efficacy of the HJ TFTs, buffer solutions and real saliva samples containing multiple metabolites were utilized. The target samples were prepared with varying concentrations of UA and 25(OH)Vit‐D3 to assess the sensitivity and specificity of the HJ‐TFTs‐based microarrays. Figure 2f demonstrates that the transfer curve of the HJ‐TFT undergoes a shift toward more negative gate voltages upon adding uric acid concentrations from 500 nm to 1000 µM. The clear threshold voltage shift (ΔV_TH_) towards more negative V_G_ is noticeable even at the low concentration of 1 µM and becomes more prominent as the concentration of UA increases up to 1000 µM. The results indicate that the electrical response of the enzyme‐modified HJ‐TFT is significantly influenced by the presence of UA in the sensing surface, which confirms its ability to detect UA across a wide range of concentrations. Similarly, the transfer curve of the HJ‐TFT exhibits a negative shift in gate voltage when Vit‐D3 concentrations increase from 100 pM to 120 nm (Figure S4b, Supporting Information). This suggests that the presence of Vit‐D3 on the sensing surface significantly modulates the electrical response of the antibody‐modified HJ‐TFT.

Uric acid sensing involves the oxidation of uric acid in the presence of uricase, a specific enzyme, which leads to the formation of allantoin and hydrogen peroxide (H_2_O_2_).^[^ ^82^, ^83^ ^]^ The subsequent electro‐oxidation of H_2_O_2_ under an applied gate voltage generates hydrogen ions (protons) and electrons, which contribute to the electrical response of the sensing channel of HJ‐TFT. The excess electrons produced by the electro‐oxidation of H_2_O_2_ under an applied gate voltage are believed to increase the carrier concentration in the In_2_O_3_/ZnO sensing channel. This alteration in carrier concentration, which resembles electronic doping, leads to a change in the drain current when UA is introduced into the sensing area of the middle channel, allowing for the selective detection of UA over a wide range of concentrations. In brief, the UA‐sensing mechanism can be described as:

(1) $$Uric\;acid+O_{2\;}\overset{Uricase}{\to }\;Allantoin+H_{2}O_{2}$$

(2) $$H_{2}O_{2}\to 2H^{+}+O_{2}+2e^{-}$$

**Figure 3:**
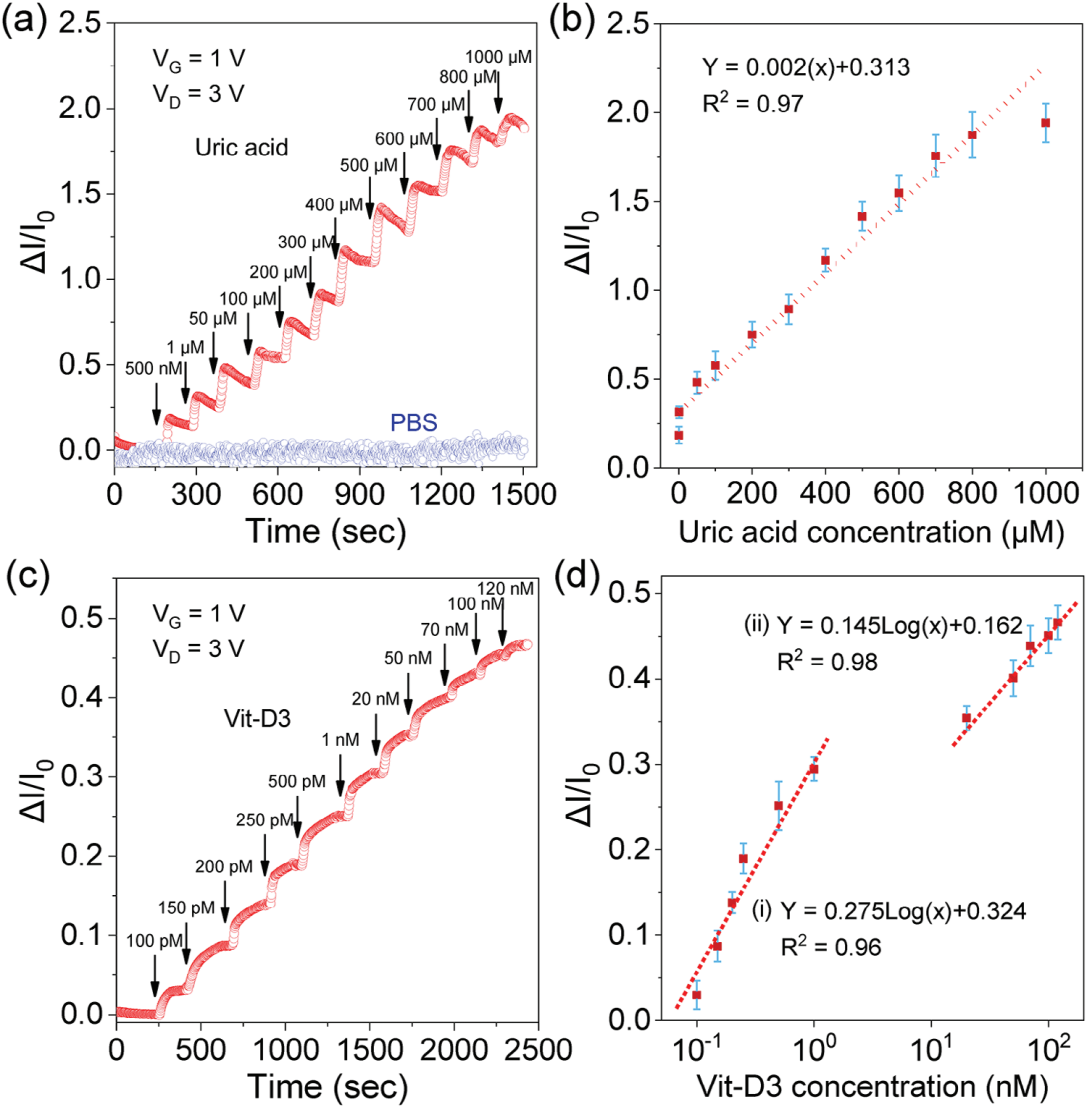
Real‐time response of HJ‐TFT microarrays measured for detections of UA and 25(OH)Vit‐D3 at constant V_G_ = 1 V, V_D_ = 3 V, a) Normalized drain current ΔI/I_0_) versus time for various concentrations of UA ranging from 500 nm to 1000 µM in 1 mM PBS. The arrow positions indicate the times at which a new UA concentration droplet was added to the middle sensing area. For comparison, the current is also measured for PBS alone without any UA in the sensing channel. b) The corresponding calibration plot of the HJ‐TFT shows the values of stabilized sensing currents versus UA concentration in a linear scale number of sensors n = 3). The dotted red line indicates the linear fitted calibration curve according to the given equation. c) ΔI/I_0_ versus time for various concentrations of Vit‐D3 100 pM to 120 nm) in PBS, d) The corresponding calibration plot of the HJ‐TFT for Vit‐D3 concentrations in a logarithmic scale n = 3).

To evaluate the UA sensor, its real‐time response to various UA concentrations in different sensing media (PBS buffer solution and human saliva) was recorded. **Figure** **3**a shows the real‐time monitoring of the sensing signal response to UA concentrations, ranging from 500 nm to 1000 µM in PBS. The I_D_ response was recorded over time while introducing a small volume (0.2 µL) of UA solution into the sensing channel, with fixed V_G_ and V_D_ at 1 V and +3 V, respectively, for each concentration. The sensing signal is the normalized change in the drain current, i.e., ΔI*/*I_0_ = (I_DS_‐I_0_ *)/*I_0_, where I_0_ is the initial drain current, and I_DS_ is the current recorded after it had stabilized when a new concentration of UA was introduced. The current response increase is assigned to be the result of UA oxidation in the presence of the uricase enzyme, which leads to an increase in n‐type charge carriers within the In_2_O_3_/ZnO channel. The ID initially increased and eventually saturated within approximately 120 s for each droplet containing an increased UA concentration. As a baseline comparison, the real‐time response to the medium alone (PBS) was also recorded (Figure 3a), and no change in current was observed in this case.

To evaluate reproducibility, UA sensing experiments were carried out for concentrations between 500 nm to 1000 µM and repeated on three different sensors (n = 3) using 1 mM PBS as the medium. The results are summarized in the calibration plot in (Figure 3b) which indicates a linear range for UA concentrations from 500 nm to 1000 µM with a high correlation coefficient (*R* ^2^ = 0.97) and a calculated sensitivity of 0.2 µA µM^−1^ cm^−2^. The range of this calibration plot covers normal and higher UA levels present in saliva as well as in blood. The calculated LOD of the UA sensor was ≈41 nm in PBS based on the calculation: 3*S_b_/m*, where *S_b_* is the standard deviation calculated from the measurements using the blank PBS, and *m* is the slope of the fitted calibration curve.

The UA sensors rely on the enzymatic reaction catalyzed by uricase enzyme, which converts UA to allantoin and H_2_O_2_. Thus the detection of H_2_O_2_ is essential for quantifying the amount of UA present in the sample. Since the enzyme‐catalyzed reaction produces H_2_O_2_, measuring the H_2_O_2_ concentration directly correlates to the UA level. The current response (I/I_0_) was recorded by successive additions of H_2_O_2_ from 0.1 µM to 1 mM in PBS (Figure S6a, Supporting Information). It can be clearly observed that the response current increases with the increasing concentration of H_2_O_2_. The corresponding calibration plot is shown in (Figure S6b, Supporting Information). It displays good linear towards H_2_O_2_ concentrations in the range of 0.1 µM to 1 mM with a correlation coefficient (*R* ^2^ = 0.97).

To test the suitability of the sensor microarray for real sample analysis, fresh saliva from a healthy volunteer was collected and used as a medium for metabolite sensing. The process for saliva collection and dilution of UA and 25(OH)Vit‐D3 concentration in saliva samples is described in the Experimental Section. UA concentration ranges with physiological significance (i.e., 500 nm–1000 µM) were prepared by adding the UA in saliva. A control measurement was first performed by pipetting saliva sample without UA onto the array with an enzyme modified sensing surface to record the background values (Figure S7, Supporting Information). The saliva sample did not lead to a significant change in drain current. In contrast, the introduction of UA in concentrations ranging from 500 nm to 1000 µM, resulted in a clear current increase for each new concentration. The real‐time response is shown in Figure S8a (Supporting Information) and the calibration plot in Figure S8b indicates that the current increased linearly with UA concentration, with a correlation coefficient *R* ^2^ = 0.95 and LOD ≈152 nm. In comparison, this result is more sensitive than previously reported methods for UA sensing in saliva (Table S1, Supporting Information). These results demonstrate that the UA sensor is capable of detecting salivary UA levels in physiologically relevant ranges.

To develop 25(OH)Vit‐D3 sensors, the modified surface of HJ‐TFTs was first incubated with the 25(OH)Vit‐D3 antibody, then different concentrations of 25(OH)Vit‐D3 were added to the antibody‐modified sensing surface, and the real‐time response was measured (Figure 3c). The current response (ΔI/I_0_) increased consistently with increasing 25(OH)Vit‐D3 concentration (ranging from 1 nm to 20 mM). The sensing mechanism of the Vit‐D3 sensor involves immobilizing a specific recombinant anti‐25(OH)Vit‐D3 antibody on the sensing surface of the device. When the liquid sample containing 25(OH)Vit‐D3 is introduced, the specific region of 25(OH)Vit‐D3 molecules bind to the antibody, forming an antigen‐antibody immunocomplex^[^ ^84^ ^]^ In this case, the epitope of the recombinant Vit‐D3 antibody [RM3] is likely to specifically bind to the area surrounding C24, C25, C26, and C27 of 25(OH)Vit‐D3 molecule. In particular, anti‐25(OH)Vit‐D3 antibody binds to the specific atom C25 on the 25(OH)Vit‐D3, it able to recognize the native structure of Vit‐D3. This interaction alters the electronic configuration of the complex and the electrostatics on the channel surface, that is directly proportional to the concentration of 25(OH)Vit‐D3 in the sample. The perturbation is then detected by the HJ‐TFT, which provides a rapid measurement of 25(OH)Vit‐D3 levels in the sample within 60 s.

Moreover, the HJ‐TFTs were employed for the determination of 25(OH)Vit‐D3 in real saliva. (Figure S9a, Supporting Information) shows the real‐time monitoring of the sensing signal response with a 25(OH)Vit‐D3 concentration in human saliva. The resulting change in current (ΔI*/*I_0_) exhibit a linear relationship with the concentrations of 25(OH)Vit‐D3 from 100 pM to 120 nm in both PBS (Figure 3d) and real saliva (Figure S9b, Supporting Information). The calibration curve of 25(OH)Vit‐D3 sensor exhibited two linear range from (100 pM to 1 nm), and (20 nm to 120 nm) with a correlation coefficient (*R* ^2^ = 0.96, *R* ^2^ = 0.98) in PBS and (*R* ^2^ = 0.96, *R* ^2^ = 0.96) in real saliva. The linear range of the sensor covers both the deficiency and normal levels of 25(OH)Vit‐D3 in both blood and saliva. The calculated LODs were ≈2 pM in PBS and ≈7 pM in real saliva, while the sensitivity was determined 27.5 µA nM^−1^ cm^−2^ in PBS, and 17.8 µA nM^−1^ cm^−2^ in saliva. This result is among the lowest when compared to the currently still few studies available where saliva is used as a medium (Table S2, Supporting Information). To our knowledge, this is the first report on the electrical detection of multiple metabolites in human saliva using solid‐state transistor microarrays.

### 2.4 Selectivity and Multi‐Analyte Detection Using Transistor Microarrays

**Figure 4:**
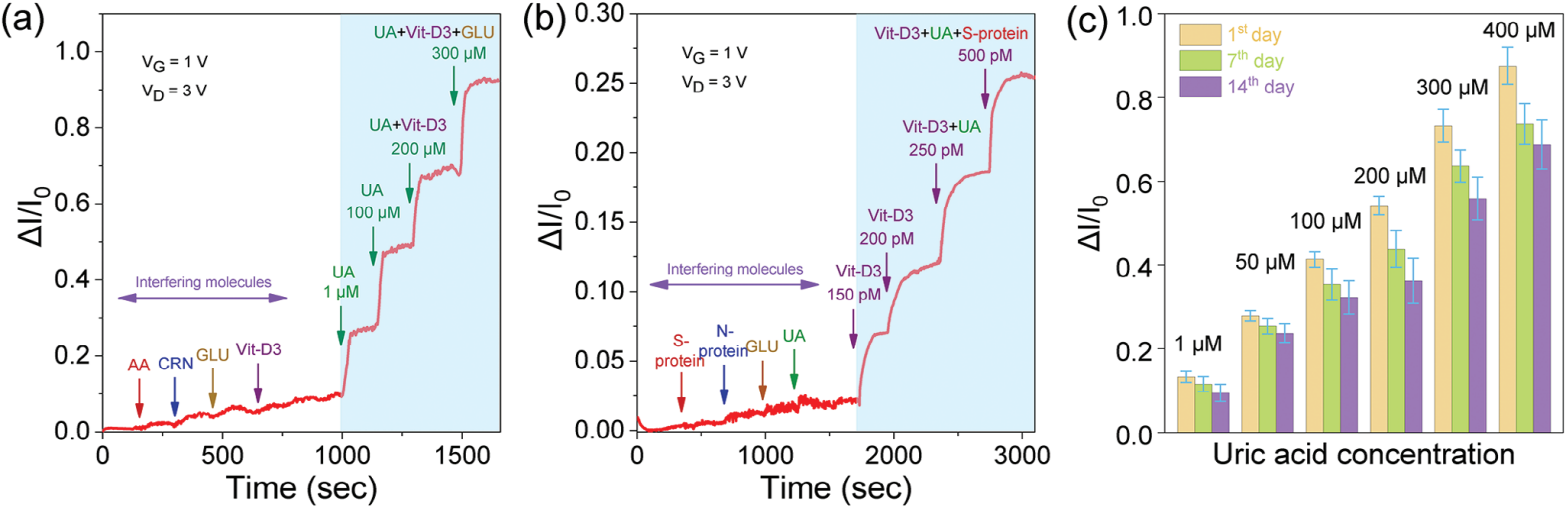
Selectivity test of the HJ‐TFT microarrays for detection of UA and Vit‐D3 in the presence of interfering molecules. a) Selectivity test of the HJ‐TFT toward UA concentration 1 µM to 300 µM) and interfering molecules AA, 10 µM), creatinine CRN, 10 µM), glucose GLU, 10 µM), Vit‐D3 500 pM) in 1 mM PBS. b) Selectivity test with a Vit‐D3 concentration 150 pM to 500 pM) and interfering analytes of Spike S) protein 500 pM), Nucleocapsid N) protein 500 pM), GLU, UA 10 µM) in 1 mM PBS. c) Stability test of the HJ‐TFT microarrays stored in ambient temperature where the current response for UA 1 µM to 400 µM) was tested on the 1st, 7th day, and 14th day after fabrication.

To evaluate the selectivity of our HJ‐TFT microarray detectors, ΔI/I_0_ was monitored during the addition of interfering molecules that are typically present in considerable amounts in biofluids. For UA detecting HJ‐TFTs where the sensing area was functionalized with uricase, the real‐time response was recorded during the introduction of interfering analytes such as ascorbic acid (AA), creatinine (CRN), glucose (GLU), Vit‐D3 as well as mixed solutions (UA+Vit‐D3) and (UA+Vit‐D3+GLU) that include UA in 1 mM PBS (**Figure** **4**a). There was no significant change for AA, CRN, GLU, and Vit‐D3, but a clear current increase for UA concentrations from 1 µM to 300 µM was detected. Additionally, the device showed a current response upon adding mixed solutions containing UA. These experiments indicate that the HJ‐TFT shows selectivity toward UA even in the presence of various interfering species.

For Vit‐D3 detecting HJ‐TFTs, selectivity tests were carried out by introducing the interfering analytes spike protein, nucleocapsid protein, GLU and UA, as well as Vit‐D3 by itself and in mixed solutions (Vit‐D3+UA) and (Vit‐D3+UA+Spike protein), each in 1 mM PBS (Figure 4b). The current response (ΔI/I_0_) consistently increased after each increasing Vit‐D3 concentration (from 150 pM to 500 pM) was added to the sensing channel, but did not change significantly when either spike protein, nucleocapsid protein, GLU, or UA were added. Moreover, the sensor was evaluated against UA and interfering analytes (AA, CRN, GLU, Vit‐D3) as well as mixed solutions (UA+Vit‐D3+Glu) in real saliva as the sensing medium (Figure S10, Supporting Information). The results indicate that the UA sensor's performance was not significantly affected when exposed to interfering analytes. However, noticeable increase in signal (ΔI/I_0_) was observed only when the modified HJ‐TFT was exposed to UA in real saliva. The results show that the proposed HJ‐TFT microarrays could achieve the specific detection of UA and 25(OH)Vit‐D3, which is attributed to the selective enzyme and antibody that are part of the biofunctionalization of the metal oxide channel.

### 2.5 Operational Stability

The stability of the HJ‐TFT biosensors was tested over two weeks. The enzyme and antibody‐modified biosensor microarrays were stored at room temperature stored under an N_2_ environment to minimize exposure to light and oxygen, which can potentially degrade the bioactivity of the enzymes or antibody. The sensor's response to UA (1 µM to 400 µM) and Vit‐D3 (100 pM to 1 nm) was recorded on the 1st day after fabrication and again after storage in ambient temperature for 7 and 14 days, respectively. Figure 4c demonstrates the changes in the current response to UA concentrations from 1 to 400 µM over time. It is apparent that the overall current signal of the sensor decreased with increasing storage duration, dropping by 8% after 7 days and by 12% on the 14th day (Figure S11a, Supporting Information), which is comparable to other reported studies.^[^ ^5^, ^8^, ^23^ ^]^ The stability of the biosensor microarray was also evaluated by measuring the relative standard deviation (RSD) values at a UA concentration of 50 µM and Vit‐D3 concentration of 200 pM (considered the low concentration in real saliva) from the 1st to the 14th day. For UA (50 µM), the real‐time response yielded a reproducibility RSD value of 4.27, 7.42 and 9.27% on 1st, 7th, and 14th day, respectively. Similarly, for the Vit‐D3 (200 pM), the real‐time response showed a good reproducibility with RSD values of 5.7, 8.95, and 12.11% on 1st, 7th, and 14th day, respectively (Figure S11b, Supporting Information). The results demonstrate the good stability and reproducibility of the biosensing microarray for UA and Vit‐D3 upon storage for two weeks. Thus, we conclude that the developed microarray biosensor remains functional after storage, highlighting their potential as biosensing platform for point‐of‐care applications.

## 3 Conclusion

We developed heterojunction metal oxide transistor (HJ‐TFT)‐based microarrays for non‐invasive detection of UA and 25(OH)Vit‐D3 in human saliva. The all‐solid‐state transistor microarrays are scalable to manufacture and exhibit robust operation under physiologically relevant conditions. The microarrays exhibit excellent transistor operation and can detect the presence of UA and 25(OH)Vit‐D3 in PBS and real saliva. Due to the transistors’ high signal amplification capabilities, the microarrays can detect UA at concentrations ranging from 500 nm to 1000 µM, and 100 pM to 120 nm for 25(OH)Vit‐D3 in under 60 s. The calculated LODs for UA and Vit‐D3 in PBS were ≈41 nm, and ≈5 pM, respectively. In the case of real saliva, the experimentally determined LOD was ≈152 nm for UA and ≈7 pM for Vit‐D3. Furthermore, the biosensing array demonstrated specific detection toward UA and 25(OH)Vit‐D3 detection enabled by selective functionalization of the individual transistors with uricase enzyme and 25(OH)D3 antibody. Critically, the sensors’ specificity was attested against different interfering molecules in the biofluid, including other metabolites and proteins. The ensuing multi‐analyte biosensing array exhibits robust performance with a fast response due to the transistors’ unique features, including its tri‐channel architecture and high electron mobility. The arrays represent an ultrasensitive, stable and highly scalable platform technology for detecting various bio/chemical analytes in POCT applications.

## 4 Experimental Section

### Materials and Reagents

Zinc oxide (ZnO, 99.99%), Indium nitrate hydrate (In(NO_3_)_3_. *x*H_2_O, 99.99%), Uric acid (UA; U2625), Uricase (from *Candida* sp., lyophilized powder, ≥2 units/mg, U0880‐1000UN), 2‐methoxyethanol (2‐ME, 99.8%), 3‐aminopropyltriethoxylsilane (APTES) solution (440140, 99%), Glutaraldehyde solution (G7776, 25% in H_2_O), L‐ascorbic acid (AA; A5960), Creatinine (C4255), β‐D‐glucose (97%), and Phosphate buffered saline (PBS, pH 7.4, 100 mM) were purchased from Sigma‐Aldrich, USA. Ammonium hydroxide (50% v/v) was procured from Alfa Aesar, USA. Ethanol absolute (20 821.330, 99.96% v/v) was purchased from VWR chemicals, France. 25‐Hydroxyvitamin D3 (ab142766), Recombinant anti‐25‐(OH) vitamin D3 antibody [RM3] (ab219464) were purchased from Abcam, USA, and used as‐obtained without further purification. SARS‐CoV‐2 Spike S1‐ recombinant protein (40591‐V08B1) was purchased from Sino Biological, China. SARS‐CoV‐2 Nucleocapsid recombinant protein (RP‐87665) was purchased from Thermo Fisher Scientific, USA. SYLGARD 184 silicone elastomer base and Curing agent were purchased from Dow chemicals, Germany. Ultrapure de‐ionized (DI) water (purified with Milli‐Q plus system, Millipore Co.) with a resistivity of ≈18 MΩ cm was used during the experiments. Standard working solutions of UA were prepared freshly by diluting its stock solution with 1 mM PBS and real saliva samples. To prepare the standard working solutions of 25(OH)Vit‐D3, stock solution of Vit‐D3 was first prepared by dissolving 1 mg of Vit‐D3 in 100 µL of absolute ethanol, followed by serial dilution in 1 mM PBS and real saliva to obtain the desired concentration range for sensing test.

### Fabrication of Heterojunction Oxide TFT Microarrays

To fabricate the metal oxide heterojunction thin‐film transistor (In_2_O_3_/ZnO HJ‐TFTs) microarrays, heavily doped Si substrates (with 50 nm thick SiO_2_) were used to produce unique a tri‐channel configuration, and the detailed fabrication process was described in a recent publications.^[^ ^46^, ^70^ ^]^ The process involved spin‐coating an In_2_O_3_ precursor solution (Indium nitrate hydrate (In(NO_3_)_3_. *x*H_2_O)) onto a cleaned Si/SiO_2_ surface, followed by annealing in ambient air. A ZnO precursor solution (Zinc oxide) was then spin‐coated onto the top of In_2_O_3_ layer and further annealed under optimized conditions. Source and drain electrodes (Al, 40 nm) were deposited by thermal evaporation through shadow masks under high vacuum. The sensing channel region and the two side channels were defined with a specific width/length ratio of W/L 2000 µm/2000 µm, and 1800 µm /100 µm, respectively.

### Surface Modification of HJ‐TFT Microarrays

The HJ‐TFT microarrays were first treated with 3‐aminopropyltriethoxylsilane (APTES) solution (2 wt.% in toluene), reacted for 15 min., followed by rinsing with toluene and annealing at 120 °C for 1 h. A glutaraldehyde (GA) linker was then applied to the APTES containing the amino (‐NH_2_) terminal groups using a solution of 2.5% (v/v) GA in DI water for 10 min, followed by rinsing with DI water and drying under N_2_ gas flow. For UA sensing, the uricase enzyme was immobilized via the GA linker using a solution (5 mg mL^−1^) in 10 mM PBS, applied for 5 h at room temperature. The enzyme‐modified microarrays were finally rinsed with PBS and DI water to remove unbound enzymes and dried under N_2_ gas prior to any further characterization. For 25(OH)Vit‐D3 sensing, recombinant anti‐25(OH)Vit‐D3 antibody was immobilized on the sensing surface of the HJ‐TFT microarrays. The immobilization process involves incubating the sensing surface with 25(OH)Vit‐D3 antibody (50 µg mL^−1^) for 5 h using APTES/GA linker chemistry, which allows the amine (─NH_2_) groups on the antibody to react with the modified sensing surface via covalent bonding. The ethanolamine (50 mM) was used to block the remaining activated binding sites to prevent the non‐specific adsorption on the sensing surface. To achieve optimal analytical performance of the HJ‐TFT microarrays for UA and Vit‐D3 detection, a systematic optimization of the immobilization concentrations was performed for both UA enzyme (0.5–10 mg mL⁻¹) and Vit‐D3 antibody (0.5–100 µg mL⁻¹) on the sensing surface and measured the drain current response with fixed concentrations of target metabolites concentrations 500 µM UA (Figure S12a, Supporting Information) and 50 nm Vit‐D3 antibody (Figure S12b, Supporting Information). A known concentration of UA (from 500 nm to 1000 µM) and 25(OH)Vit‐D3 (from 100 pM to 120 nm) was introduced into a buffer solution and a saliva sample to achieve the desired concentration from the stock solution.

### Detection of UA and 25(OH)Vit‐D3 using HJ‐TFT Microarrays

The microarrays were exposed to various concentrations of target analytes. The respective target analyte was serially diluted from high concentration to low concentration. Then, small volume (0.2 µL) of buffer solution and human saliva were incorporated on modified microarrays encapsulated via polydimethylsiloxane (PDMS) well for control test. Next, the target solution was incorporated on modified microarrays via PDMS well from low to high concentration. The transfer characteristic (channel current (I_DS_)*_,_* versus gate voltage (V_G_)) was measured before and after the target analyte interaction under a fixed drain voltage (*V_D_*) of 3 V and by sweeping the V_G_ (−10 V to 20 V). The V_G_ shifts induced by the analytes were used as a sensing signal. For real‐time sensing applications, the channel current as a function of time was monitored by applying the respective analytes on modified microarrays under the constant bias of V_D_ = 3 V and V_G_ = 1 V. All electrical measurements were performed using three micro‐positioners (EB‐700, EVERBEING), a probe station and an Keysight B2912A Precision Source/Measure Unit at room temperature.

### Saliva Collection

Before saliva collection, the healthy volunteers were asked to fast overnight without drinking or eating anything (except water). The saliva sample (1 mL) was obtained after rinsing the mouth several times with drinking water, then holding saliva in the mouth without or minimized swallowing and then directly collecting it in an Eppendorf tube to avoid contamination.^[^ ^85^ ^]^ The sample was filtered to remove large biomolecules such as mucins using 0.2 µm pore size Whatman syringe filter (6870‐1302). The pH of the filtered saliva was measured to be in the range of 6 to 7. All protocols and procedures for using human saliva were approved by the KAUST Institutional Biosafety and Bioethics Committee (IBEC) under project number 22IBEC056.

### Statistical Analysis

The quantitative analysis results of were obtained from three individual TFT microarrays (n = 3). The sensing signal was the normalized change in the drain current, i.e., ΔI*/*I_0_ = (I_DS_‐I_0_ *)/*I_0_, where I_0_ was the initial drain current, and I_DS_ was the current recorded after it had stabilized when a new concentration of target metabolites was introduced. The calculated LOD of the UA and Vit‐D3 sensor was based on the calculation: 3*S_b_/m*, where *S_b_* was the standard deviation calculated from the measurements using the blank buffer solution, and *m* was the slope of the fitted calibration curve. The standard deviation of calibration plot were based on three individual TFT microarrays (n = 3). The data were presented as mean values ± standard deviations. Data plot and analysis of electrical measurements were carried out using Origin (OriginLab, 2019) software.

## Conflict of Interest

The authors declare no conflict of interest.

## Supporting information

Supporting Information

## Data Availability

The data that support the findings of this study are available from the corresponding author upon reasonable request.
