# Supplementary material for: Label‐Free Metal‐Oxide Transistor Biosensors for Metabolite Detection in Human Saliva
Source: Adv Sci (Weinh). 2024 Feb 21;11(27):2306038. doi: 10.1002/advs.202306038 (PMC11251559; doi:10.1002/advs.202306038)
Supplement: Supplementary file 1 — Supporting Information [file ADVS-11-2306038-s001.pdf]

ADVANCED SCIENCE
Open Access

Supporting Information

for *Adv. Sci.*, DOI 10.1002/advs.202306038

Label-Free Metal-Oxide Transistor Biosensors for Metabolite Detection in Human Saliva

Abhinav Sharma, Hendrik Faber, Wejdan S. AlGhamdi, Dipti Naphade, Yen-Hung Lin, Martin Heeney and Thomas D. Anthopoulos**

Supporting Information

Label-free Metal-oxide Transistor Biosensors for Metabolite Detection in Human Saliva

Abhinav Sharma[1], Hendrik Faber[1], Wejdan S. AlGhamdi[1], Dipti Naphade[1], Yen-Hung Lin[2], Martin Heeney[1], Thomas D. Anthopoulos[1*]*

[1]King Abdullah University of Science and Technology (KAUST), KAUST Solar Center (KSC), Thuwal 23955-6900, Saudi Arabia
[2]Department of Electronic and Computer Engineering, The Hong Kong University of Science and Technology, Clear Water Bay, Kowloon, Hong Kong

Correspondence (*): thomas.anthopoulos@kaust.edu.sa; abhinav.sharma@kaust.edu.sa

Surface characterization

The sensing surface (In_2O_3/ZnO) was characterized by atomic force microscopy (AFM, Bruker's Dimension Icon) for topological imaging and to determine average surface roughness after each modification. Additional techniques included the static water contact angle (SCA, DSA100) measurements for surface wettability and hydrophobicity, as well as X-ray photoelectron spectroscopy (XPS, Kratos Analytical, Amicus, Al Kα X-ray source) for chemical analysis of the sensing surface. For SCA, the sessile drop method was used and the contact angle was measured 5 s after a 5 μL water droplet was deposited onto each respective surface.

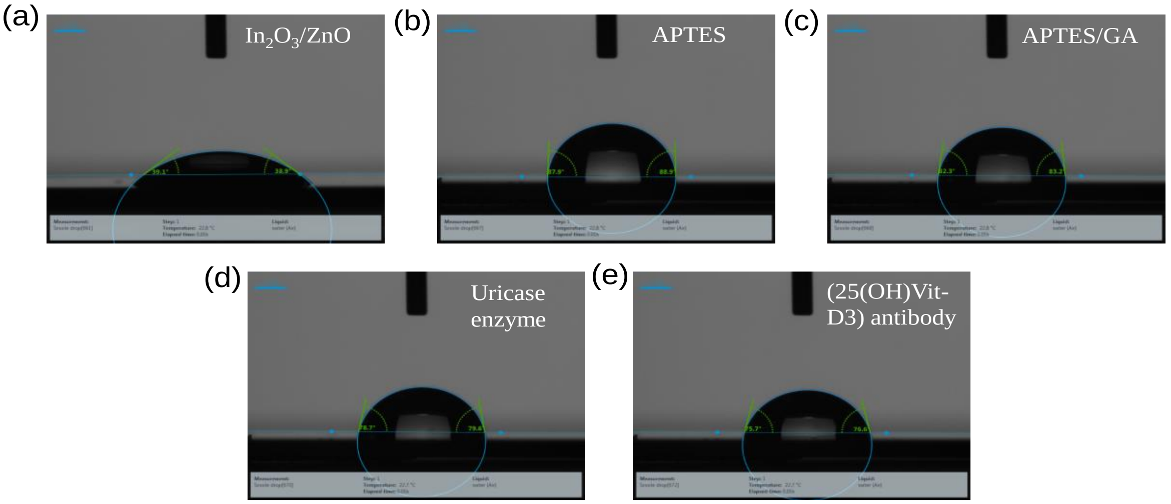

Figure S1. Water contact angle images of different functionalized surfaces: (a) In_2O_3/ZnO, (b) APTES, (c) APTES/GA, (d) APTES/GA/Uricase enzyme, (e) APTES/GA/(25(OH)Vit-D3) antibody.

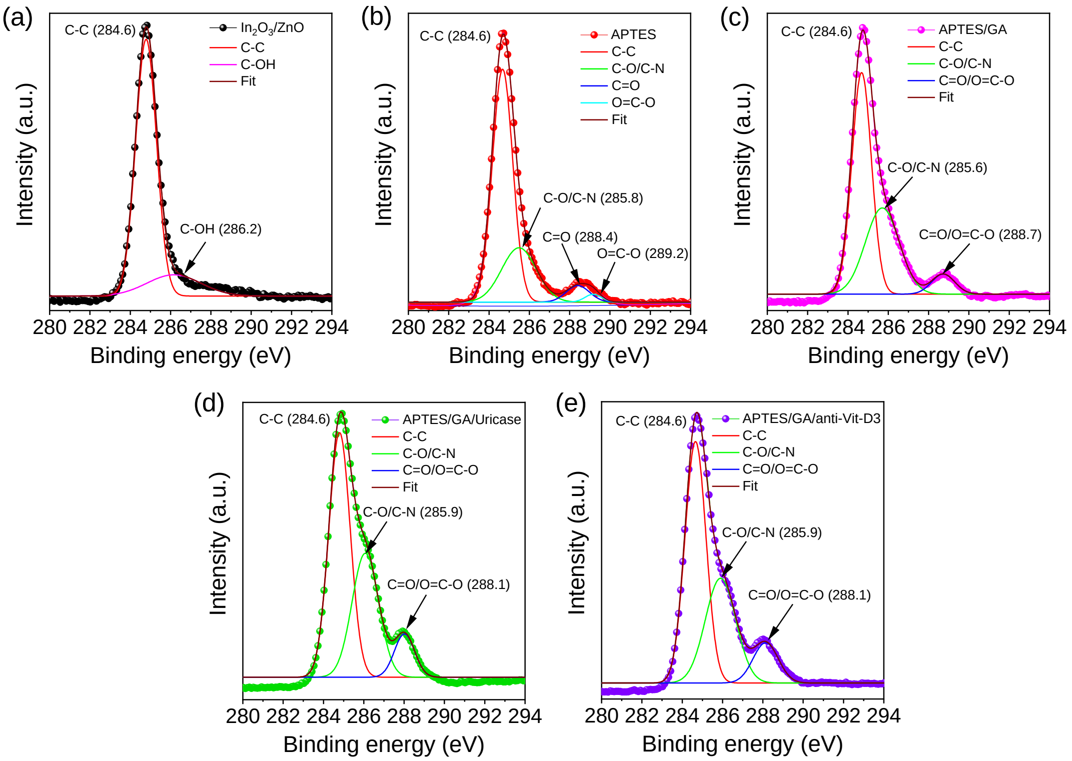

Figure S2. Fitted C1s XPS spectra of (a) In_2O_3/ZnO before and after each modification with (b) APTES, (c) APTES/GA, (d) APTES/GA/Uricase enzyme, (e) APTES/GA/(25(OH)Vit-D3) antibody.

Table S1. Comparison of the analytical performance of different UA sensors in non-invasive fluids or buffer solutions reported in the literature.

**Sensing materials**	**Biofunctionalization**	**Sensing type**	**Detection range**	**Sensitivity (S)**	**Limit of detection (LOD)**	**Biofluid**	**Response time**	**Reference**
Filter paper/Laser-induced graphene	Non-enzymatic	Differential pulse voltammetry (DPV)	10 – 300 µM (PBS)	0.363 µAcm^{-2} µM^{-1}	3.97 µM (PBS)	Urine	–	[1]
Boron-doped graphene quantum dots anchored to carbon nanotubes (B-GQDs/CNTs)	Non-enzymatic	DPV	5 – 50 µM (PBS, Sweat)	8.92 µA µM^{-1} cm^{-2} (PBS). 35.1 µA µM^{-1} cm^{-2} (Sweat)	0.99 µM	Sweat	–	[2]
Polyolefin substrate/Graphene–gold nanopaerticles (Gr–Au NPs)	Non-enzymatic	DPV	20 – 500 µM	–	1.47 µM	Artificial cerebrospinal fluid	–	[3]
Carbon fiber paper/MXene /molybdenum disulfide (MoS_2)	Non-enzymatic	DPV	0.5 – 1000 µM (PBS)	–	0.38 µM (PBS)	Urine	–	[4]
Polyimide substrate /Laser-induced graphene	Non-enzymatic	DPV	1 – 200 µM	3.50 µA µM^{-1} cm^{-2}	0.74 µM	Sweat	–	[5]
PET/Carbon veil-based electrode	Non-enzymatic	Linear sweep voltammetry (LSV)	0.09 – 700 µM (PBS)	–	0.05 µM (PBS)	Saliva	–	[6]
Polyimide/ Laser-induced graphene/ Prussian Blue nanoparticles (PBNPs)	Molecularly imprinted polymer (MIP)	LSV	10 – 320 µM	–	10 µM	Sweat	–	[7]
Filter paper/Carbon ink/Graphene quantum dots (GQDs)	Non-enzymatic	Square wave voltammetry (SWV)	0.01 – 3 µM (PBS)	–	8.4 nM (PBS)	Urine	–	[8]
Filter paper	Fe^{3+} and 1,10-phenanthroline	Colorimetric	300 – 3000 µM		98 µM	Urine	–	[9]
Glove/Carbon ink/carboxyl functionalized multiwall	Non-enzymatic	Chronoamperometric	0 – 2100 µM (PBS), 10 – 40 µM	–	3.58 µM (PBS)	Sweat	60 sec	[10]

carbon nanotubes (COOH-MWCNT)			(Sweat)					
PET/Prussian-blue-graphite ink	Uricase	Chronoamperometric	50 µM – 1000 µM (Artificial saliva), 200 µM – 1000 µM (Saliva)	2.32 µA/mM (Artificial saliva), 1.08 µA/mM (Saliva)	50 µM (Artificial saliva), 200 µM (Saliva)	Saliva	60 sec	[11]
Polyethylenet erephthalate (PET)/Carbon ink/MWCNTs	Uricase	Chronoamperometric	100 – 800 µM (PBS), 5 – 1000 µM (Artificial saliva), 200 – 1000 µM (Saliva)	–	0.33 µM (Artificial saliva)	Saliva	2 min	[12]
Printed circuit board (PCB)/Cr/Au	Uricase	Chronoamperometric	100 – 500 µM (PBS)	4.6 µA/mM	100 µM	Saliva	150 sec	[13]
In_2O_3/ZnO	Uricase	HJ-TFT microarrays	0.5 – 1000 µM (PBS, Real saliva)	0.2 µA μM^{-1} cm^{-2} (PBS), 0.1 µA μM^{-1} cm^{-2} (Real saliva)	~41 nM (PBS), ~152 nM (Real saliva)	Saliva	<60 sec	This work

Table S2. Comparison of the analytical performance of different 25(OH)Vit-D3 sensors in non-invasive fluids or buffer solutions reported in the literature.

**Sensing materials**	**Biofunctionalization**	**Sensing type**	**Detection range**	**Sensitivity (S)**	**Limit of detection (LOD)**	**Biofluid**	**Response time**	**Reference**
GCE/rGO/MoS_2	Aptamer	DPV	0.25 – 375 nM	–	0.05 nM, (PBS) 1.5 nM (Saliva)	Saliva	–	[14]
Glycol-modified polyethylene terephthalate (PETG) sheets/Au	Antibody	Chronoamperometric	125 – 500 nM	–	72.5 nM (PBS), 30 nM (Saliva)	Saliva	25 min	[15]
Polyimide/ Laser-induced graphene/ Prussian Blue nanoparticles (PBNPs)	Molecularly imprinted polymer (MIP)	LSV	10 – 320 µM	–	10 µM	Sweat	–	[7]
In_2O_3/ZnO	Antibody	HJ-TFT microarrays	0.1 – 120 nM (PBS, Real saliva)	27.5 µA nM^{-1} cm^{-2} (PBS), 17.8 µA nM^{-1} cm^{-2} (Real saliva)	~2 pM (PBS), ~7 pM (Real saliva)	Saliva	<60 sec	This work

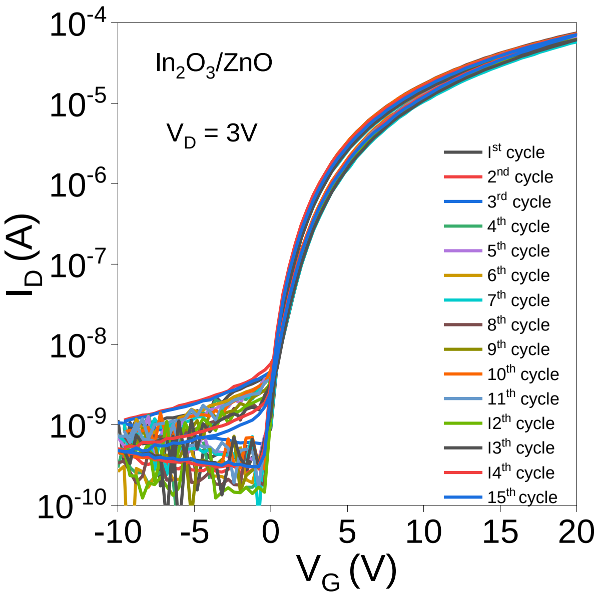

Figure S3 Transfer characteristics of 15 repeated cycles measured on a single tri-channel oxide HJ-TFT device showing the operational stability.

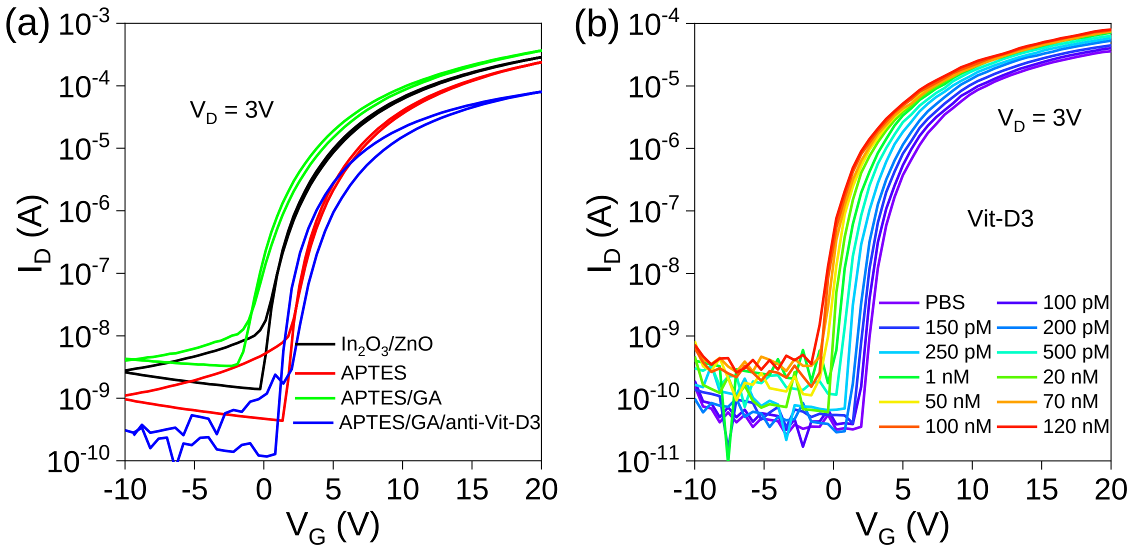

Figure S4. (a) Transfer characteristics of the In_2O_3/ZnO HJ-TFT microarray measured before and after each functionalization with APTES, APTES/GA, and APTES/GA/anti-25(OH)Vit-D3 antibody. (b) Transfer characteristics (forward sweeps) for a HJ-TFT measured at V_D = 3V in the

presence of a buffer solution (1 mM PBS) containing uric acid at different concentrations (100 pM to 120 nM).

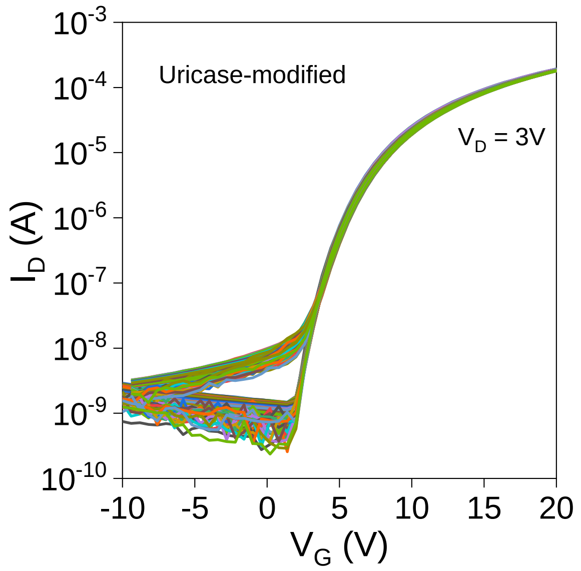

Figure S5. Multiple transfer characteristics (120 forward and reverse sweeps) of a HJ-TFT following immobilization of the uricase enzyme on the surface of the sensing channel.

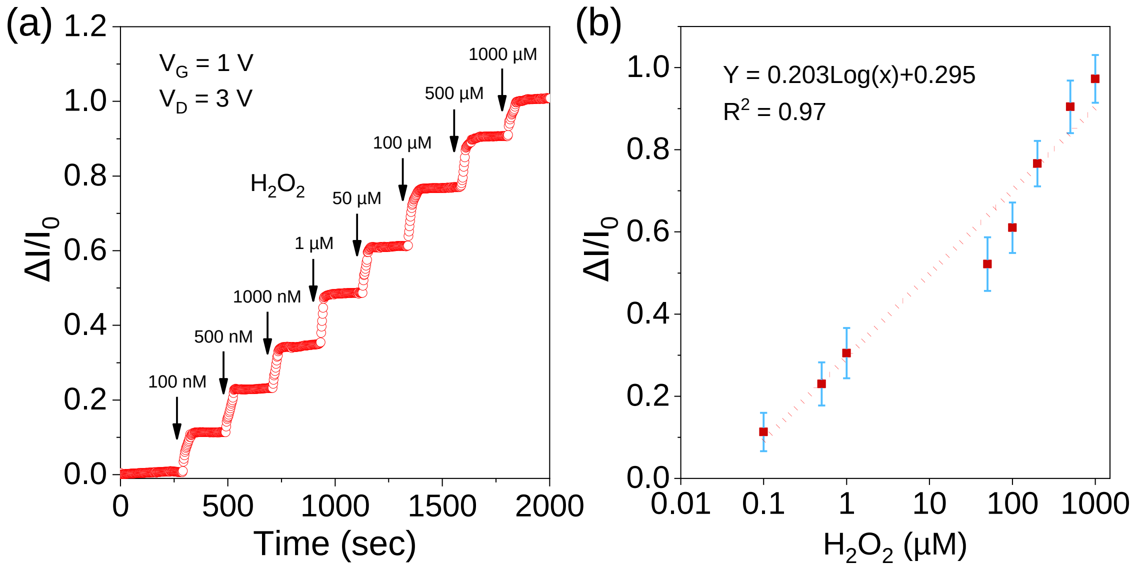

Figure S6. (a) Normalized drain current ($\Delta I/I_0$) vs time for various concentrations of H_2O_2 ranging from 0.1 to 1 mM in 1 mM PBS. (b) The corresponding calibration plot of the HJ-TFT for H_2O_2 concentrations in a logarithmic scale (n = 3).

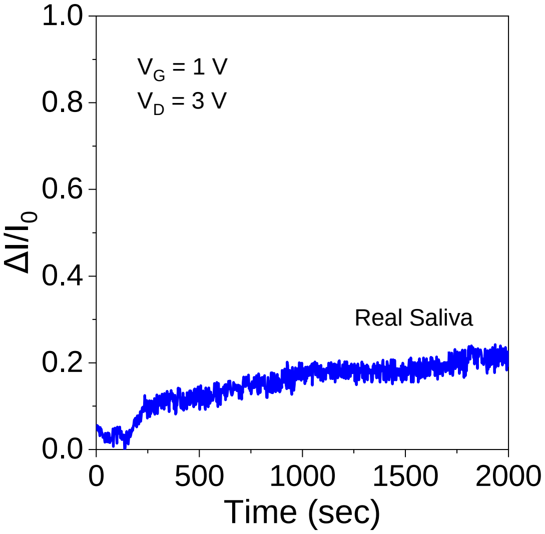

Figure S7. Normalized drain current ($\Delta I/I_0$) vs time response was recorded by adding real saliva sample without target analytes at constant V_G = 1V, V_D = 3V.

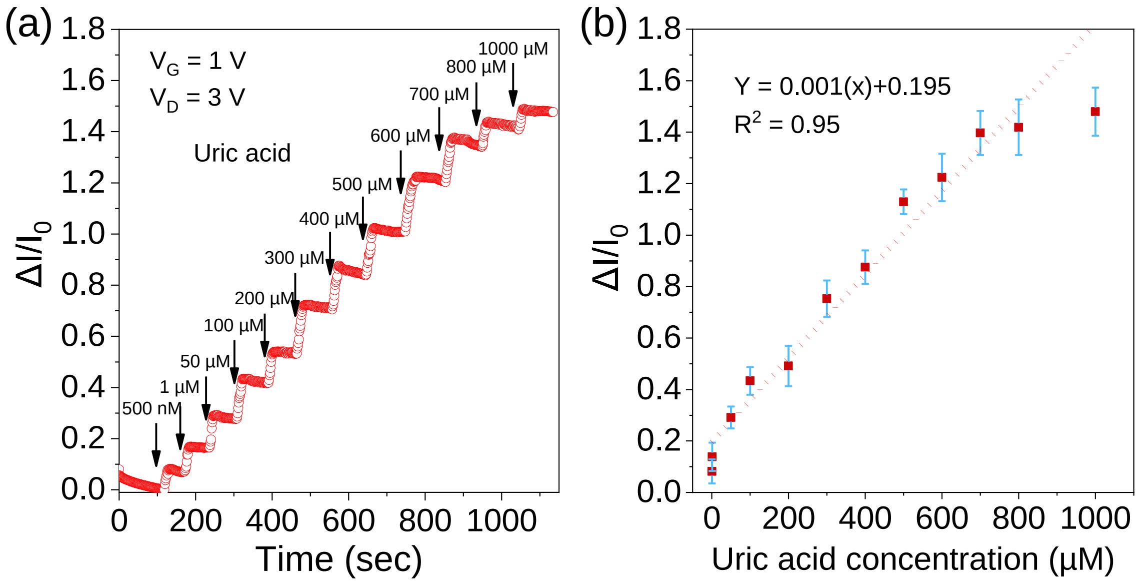

Figure S8. (a) Normalized drain current ($\Delta I/I_0$) vs time for various UA concentrations (500 nM to 1000 μM) in real saliva at constant V_G = 1V, V_D = 3V. (b) The corresponding calibration plot of HJ-TFTs (n=3) shows stabilized sensing current vs UA concentration in the linear scale.

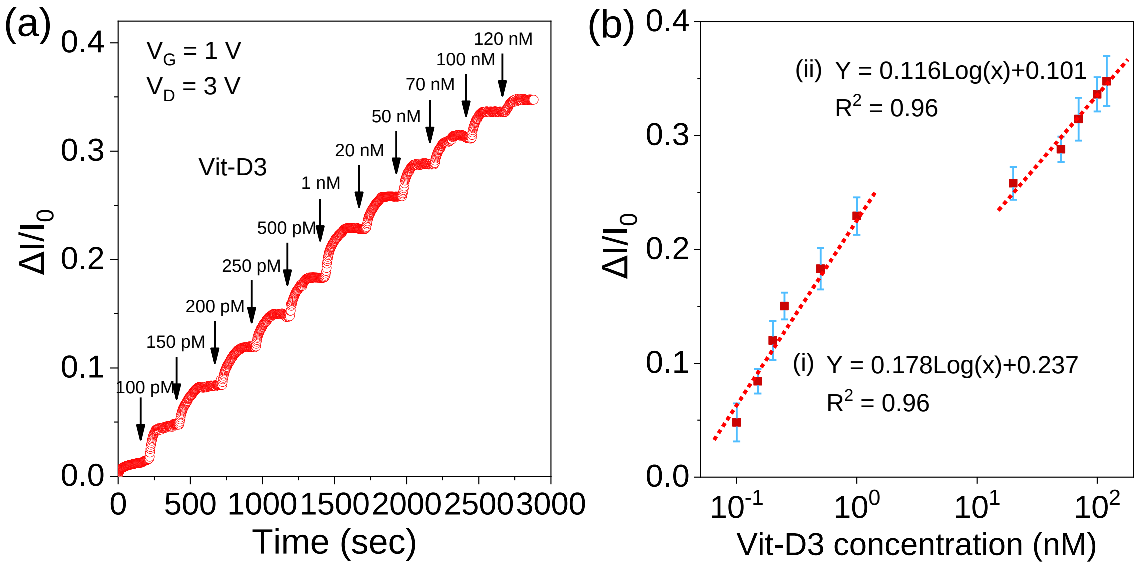

Figure S9. (a) Normalized drain current ($\Delta I/I_0$) vs time for various 25(OH)Vit-D3 concentrations (100 pM to 120 nM) in real saliva at constant V_G = 1V, V_D = 3V. (b) The corresponding calibration plot of HJ-TFTs (n=3) shows stabilized sensing current vs 25(OH)Vit-D3 concentration in the logarithmic scale.

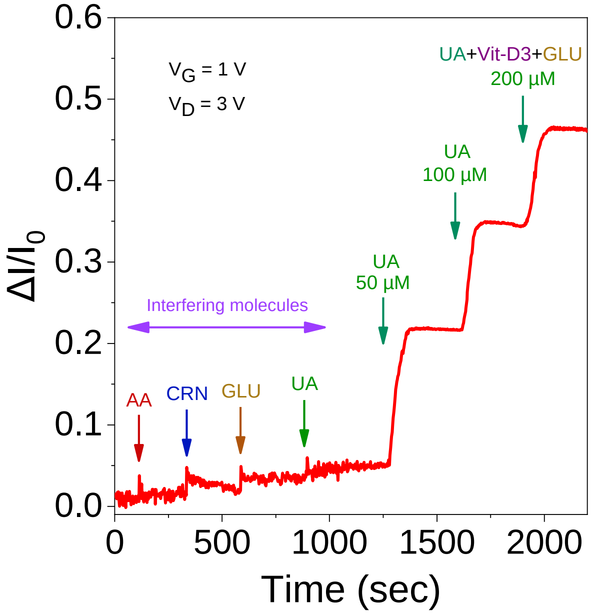

Figure S10. Selectivity test of the HJ-TFT toward UA concentration (50 μM to 200 μM) with interfering molecules (AA, CRN, GLU, Vit-D3) and mix solution of UA+Vit-D3+Glu in real saliva.

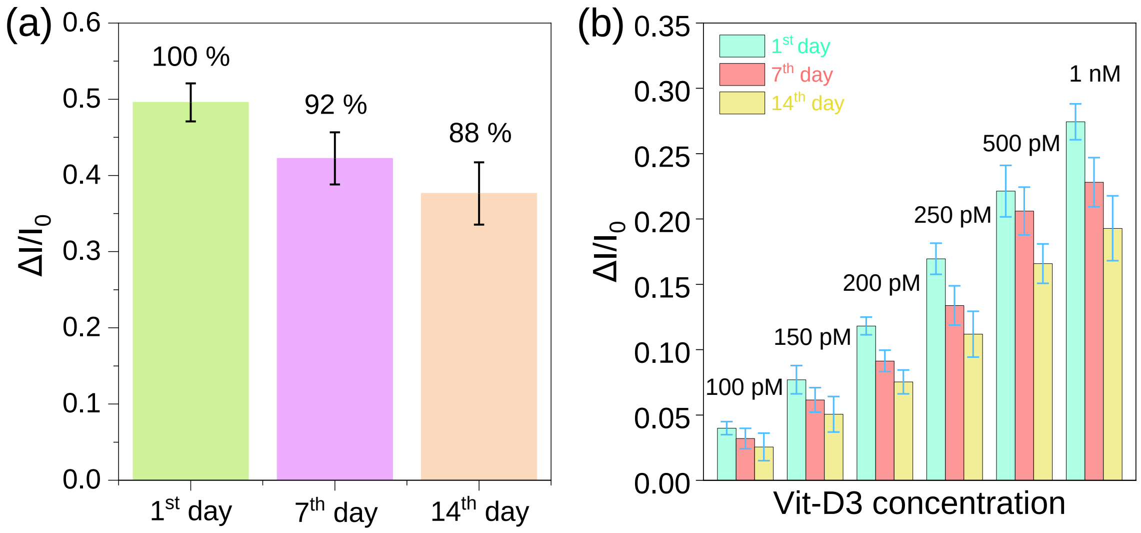

Figure S11. (a) The average value of normalized drain current ($\Delta I/I_0$) of the HJ-TFT biosensors stored in the ambient atmosphere for 1, 7 and 14 days. (b) Stability test of the HJ-TFT microarrays stored in ambient temperature where the current response for Vit-D3 (100 pM to 1 nM) was tested on the 1st, 7th day, and 14th day after fabrication.

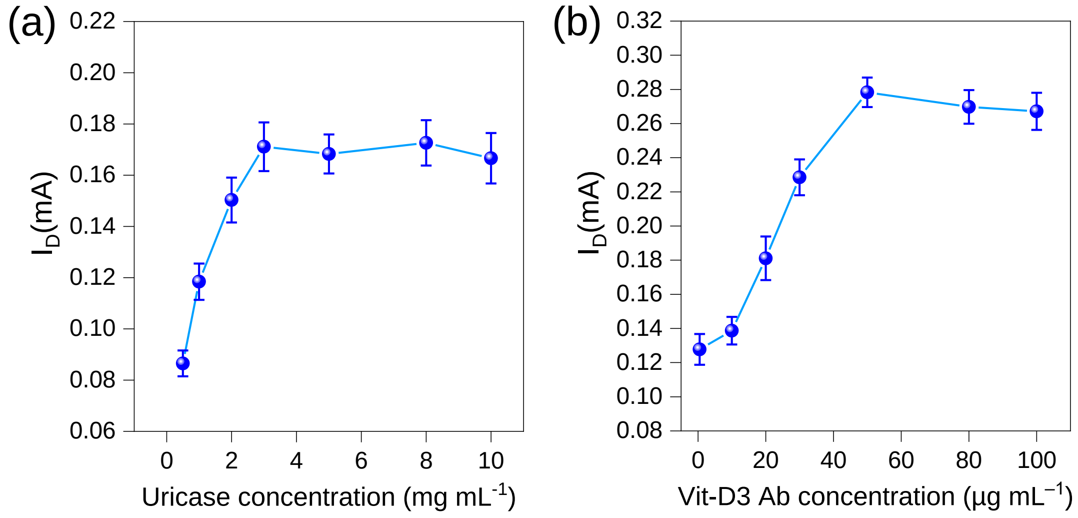

Figure S12. The drain current (V_G = -10 to 20V, V_D = 3V) of UA and Vit-D3 detectors using varied immobilization concentration of, (a) UA enzyme (0.5 mg mL^{-1} to 10 mg mL^{-1}) and (b)Vit-D3 antibody (0.5 μg mL^{-1} to 100 μg mL^{-1}) modified HJ-TFT on sensing surface. The error bar is calculated from the responses of at least three devices.

References

[1] B. Kulyk, S. O. Pereira, A. J. Fernandes, E. Fortunato, F. M. Costa, N. F. Santos, *Carbon* **2022**, 197, 253.

[2] Y.-X. Wang, M. Rinawati, J.-D. Zhan, K.-Y. Lin, C.-J. Huang, K.-J. Chen, H. Mizuguchi, J.-C. Jiang, B.-J. Hwang, M.-H. Yeh, *ACS Appl. Nano Mater.* **2022**, 5, 11100.

[3] W. He, X. Ye, T. Cui, *IEEE Sensors Journal* **2021**, 21, 26556.

[4] J. Zhao, C. He, W. Wu, H. Yang, L. Peng, L. Wen, Z. Hu, C. Hou, D. Huo, *Chemical Engineering Journal* **2022**, 446, 136841.

[5] Y. Yang, Y. Song, X. Bo, J. Min, O. S. Pak, L. Zhu, M. Wang, J. Tu, A. Kogan, H. Zhang, *Nat. Biotechnol.* **2020**, 38, 217.

[6] M. A. Bukharinova, N. Y. Stozhko, E. A. Novakovskaya, E. I. Khamzina, A. V. Tarasov, S. V. Sokolkov, *Biosens* **2021**, 11, 287.

[7] M. Wang, Y. Yang, J. Min, Y. Song, J. Tu, D. Mukasa, C. Ye, C. Xu, N. Heflin, J. S. McCune, *Nat. Biomed. Eng.* **2022**, 6, 1225.

[8] F. H. Cincotto, E. L. Fava, F. C. Moraes, O. Fatibello-Filho, R. C. Faria, *Talanta* **2019**, 195, 62.

[9] E. L. Rossini, M. I. Milani, E. Carrilho, L. Pezza, H. R. Pezza, *Anal. Chim. Acta* **2018**, 997, 16.

[10] Z. Li, Y. Wang, Z. Fan, Y. Sun, Y. Sun, Y. Yang, Y. Zhang, J. Ma, Z. Wang, Z. Zhu, *Biosens* **2023**, 13, 105.

[11] J. Kim, S. Imani, W. R. de Araujo, J. Warchall, G. Valdés-Ramírez, T. R. Paixão, P. P. Mercier, J. Wang, *Biosens. Bioelectron.* **2015**, 74, 1061.

[12] W. Shi, J. Li, J. Wu, Q. Wei, C. Chen, N. Bao, C. Yu, H. Gu, *Anal. Bioanal. Chem.* **2020**, 412, 7275.

[13] Z. Liu, Y. Chen, M. Zhang, T. Sun, K. Li, S. Han, H.-J. Chen, *Biosens* **2021**, 11, 242.

[14] J. Park, M. Kim, W. Kim, S. Jo, W. Kim, C. Kim, H. Park, W. Lee, J. Park, *Sens. Actuators B Chem.* **2022**, 355, 131239.

[15] V. R.-V. Montiel, J. R. Sempionatto, E. Vargas, E. Bailey, J. May, A. Bulbarello, A. Düsterloh, N. Matusheski, J. Wang, *Biosens. Bioelectron.* **2021**, 194, 113590.
